# Optimization of Molecular Dynamics Simulations of c-MYC^1-88^—An Intrinsically Disordered System

**DOI:** 10.3390/life10070109

**Published:** 2020-07-10

**Authors:** Sandra S. Sullivan, Robert O.J. Weinzierl

**Affiliations:** Department of Life Sciences, Imperial College London, London SW7 2AZ, UK; s.sullivan15@imperial.ac.uk

**Keywords:** oncoproteins, c-MYC, molecular dynamics simulations

## Abstract

Many of the proteins involved in key cellular regulatory events contain extensive intrinsically disordered regions that are not readily amenable to conventional structure/function dissection. The oncoprotein c-MYC plays a key role in controlling cell proliferation and apoptosis and more than 70% of the primary sequence is disordered. Computational approaches that shed light on the range of secondary and tertiary structural conformations therefore provide the only realistic chance to study such proteins. Here, we describe the results of several tests of force fields and water models employed in molecular dynamics simulations for the N-terminal 88 amino acids of c-MYC. Comparisons of the simulation data with experimental secondary structure assignments obtained by NMR establish a particular implicit solvation approach as highly congruent. The results provide insights into the structural dynamics of c-MYC^1-88^, which will be useful for guiding future experimental approaches. The protocols for trajectory analysis described here will be applicable for the analysis of a variety of computational simulations of intrinsically disordered proteins.

## 1. Introduction

Intrinsically disordered proteins (IDPs) exhibit vastly different structural dynamics as compared to folded proteins. Instead of folding predictably, according to their amino acid sequence, IDPs exist as a rapidly changing ensemble of conformations. This structural diversity allows them to bind multiple interaction partners, and places them at the center of key cellular pathways [1]. Their structural features, and ever-changing conformational dynamics, are based on their unusual amino acid composition. IDPs are enriched in polar and charged residues and depleted in hydrophobic amino acids required for the formation of stable cores. This destabilizes the protein fold and allows IDPs to change rapidly between a wide range of alternate conformations [2]. The greatest challenge encountered when studying IDPs is to identify methods that describe their rich dynamic complexity. Conventionally applied structural methods, such as X-ray crystallography and cryo-electron microscopy, are unsuitable to study IDPs—these proteins do not form crystals and inhabit a vast number of conformations which cannot be represented adequately by a small number of high-resolution structural models. Nuclear magnetic resonance (NMR) techniques capture ensemble-averaged data but fail to deliver the detailed structural models that are required, for example, to identify the formation of transient pockets for drug-binding studies. Recently, in silico methods such as molecular dynamics (MD) simulations have emerged as promising alternatives for exploring the conformations and dynamical aspects of IDPs. Nowadays, MD simulations can be run efficiently on workstations using graphics processing units (GPUs), which permits low-cost simulation parallelization [3,4]. The understanding of the physicochemical basis underpinning the biological system simulation estimates has also improved notably [5]. The accuracy of MD simulations depends greatly on precise parameterization. Specifically, the widely used AMBER and CHARMM force field refinements, in conjunction with a variety of water models, have produced accurate descriptions of small globular proteins [6]. Force field and water models display, however, certain shortcomings when it comes to the MD simulations of IDPs. The older AMBER ff99 and CHARMM22/CMAP force fields tend to overestimate α-helical content, whilst the more recent AMBER ff99SB version underestimates it. GROMOS96 displays a consistent bias towards the artefactual creation of β-sheet structures [7,8]. Recent attempts at improving the accuracy of standard force fields, such as AMBER ff99SB-ILDN, AMBER ff99SBNMR1-ILDN, GROMOS 53A6 and GROMOS 54A7, still produce excessively collapsed proteins that fail to emulate the structural diversity associated with IDPs and/or exhibit a considerable bias towards folded secondary structure motifs [9]. The recent CHARMM36 force field displayed a marked bias towards left-handed α-helix oversampling, which was addressed by their latest iteration for IDP simulation—CHARMM36m [10]. However, for AMBER, the simulation package used in this paper, even one of its latest force field releases, ff14SB, touted to have improved sampling accuracy of backbone and sidechain geometry, was found to create overly compacted structures displaying excessive α-helical and/or β-sheet formations [11,12].

Broadly, two main strategies have been proposed to correct the biases and limitations of conventional MD simulation parameters: to re-design the simulation models by optimizing the force fields or improve the accuracy of the simulation by enhancing the solvation conditions [13].

### 1.1. Optimized Force Fields for IDP Simulations

In 2017, two novel AMBER force field modifications for IDP simulation were published: ff14IDPs [14] and ff14IDPSFF [15]. These re-parameterize AMBER ff14SB but retain most of its main features. The ff14IDPs force field iteration improves IDP sampling by taking advantage of the unusual amino acid composition of IDPs through modification of the φ/ψ distributions of eight disorder-promoting amino acids (G, A, S, P, R, Q, E and K). The backbone dihedral terms for all 20 naturally occurring amino acids are upgraded with ff14IDPSFF. A recent study demonstrated that the ff14IDPSFF force field improves the accuracy of MD simulations for at least a subset of IDPs [16].

### 1.2. Optimizing Explicit and Implicit Water Models

IDPs are very susceptible to the type of solvation model used to generate the simulation environment. Due to the large solvent-exposed surfaces of IDPs, they are highly responsive to protein–water interaction forces. Incorrect solvation potentials have been recognized as a major source of inaccurate, overly stabilized, or fragmentary IDP conformational description [1]. In conventional simulations of globular proteins, AMBER and CHARMM force fields (and their variants) are frequently used in conjunction with the TIP3P water model as the explicit solvation standard, whilst GROMOS applies the SPC model [17]. To address the conventional solvation methods bias, CHARMM36m proposed key Lennard-Jones modifications to the TIP3P water hydrogens, suggesting that the optimization of the water hydrogen atom dispersion terms, instead of the oxygen atoms, leads to more balanced protein–water interactions [10]. Other solvation models, such as TIP4P-D, have been developed to counteract specifically the compaction effects of TIP3P and SPC [13]. Even modest increases in London dispersion interactions between the protein and solute were found to improve the sampling of the unfolded states of IDPs [12]. In TIP4P-D, the overall tendency for intramolecular interactions within proteins is reduced in favor of increased protein–water interactions. The application of the TIP4P-D water model expands the conformational diversity of the disordered states of IDPs and was found to be in excellent agreement with experimental small-angle X-ray scattering (SAXS) data [18].

In contrast to the explicit solvation methods discussed above, implicit solvent models represent solvation-free energy as a continuum of electrostatic approximation. At each simulation point, the solvation potential of the system is re-computed based only on the degrees of freedom of the coordinates of the solute [19]. Implicit models naturally lead to enhanced macromolecular conformational sampling due to the lack of viscosity associated with the explicit water environment [19]. Despite some of these advantages, continuum-implicit solvation methods have largely been neglected for MD simulations. This is mostly due to concerns that implicit solvation strategies compromise accuracy [6].

### 1.3. Assessing Simulation Convergence

The concept of convergence in MD simulations is elusive but critical. It is important to ensure that meaningful transitions are being observed in simulations, and that the conformational space is sampled in a statistically meaningful manner. In MD simulations, convergence is seen as the plateauing of observables for an adequately long time [20]. Several methods have established themselves as useful for analyzing an ensemble of conformations to represent equilibrium [21]. These include assessing the evolution of observables in terms of intra-molecular interaction energy, hydrogen bonding, root mean square fluctuations (RMSFs) and torsion angle evolution [22]. Additionally, cluster analysis and principal component analysis (PCA) have played a major role in assessing the structural diversity generated in MD simulations [23,24,25]. Despite several metrics being used, the most ubiquitous and standard technique for convergence determination is the calculation of the root mean square deviation (RMSD) over the course of the trajectory [26]. Such methods, however, perform poorly for predicting conformational convergence if the conformational ensemble differs largely and constantly from the reference structure (as in the case of IDP simulations).

Here, we assess the MD simulations of two types of model IDPs by comparing conformations created by them with an extensively sampled probability distribution of protein conformations created by Monte Carlo (MC) simulations using a Markov chain to systematically generate random system conformations dependent on the prior state [27]. Such a Markov chain Monte Carlo (MCMC) approach for protein inference creates stochastic samples from the Boltzmann distribution to approximate the converged target distribution [28]. Thus, comparing the well-sampled probability distribution approximated by the MCMC simulations to the conformational landscape derived from the MD simulations provides another method to infer convergence that is better suited for IDP simulations.

## 2. Materials and Methods

### 2.1. MD Set-up

All MD simulations were created using the AMBER16 MD simulation package [29]. For testing the various force fields (ff14SB [30]; ff14IDPs [14]; ff14IDPSFF [15]), simulations were parameterized and solvated with TIP3P water using tLEaP (AmberTools 16; [30]). A periodic water box was created with a 15 Å distance between the molecules and the limits of the box. The solvation environment was enriched with Na^+^ and Cl^−^ ions to a final concentration of 150 mM NaCl. The explicitly solvated simulations started with a conventional molecular dynamics (cMD) simulation that included two successive minimizations: Min1 consisted of a solvent minimization run with the protein fixed, 10,000 maximum cycles and 5000 ncycles of steepest descent; Min2 aimed for a total system minimization with 2500 maximum cycles and 1000 ncycles of steepest descent. Both had a 10 Å nonbonded cutoff. This was followed by two production runs: Md1 entailed 100 ps of MD with weak restraints on the protein, with SHAKE to perform hydrogen bond length constraints, no force evaluation for bonds with hydrogen and the system was kept at a constant volume, to reach a temperature of 310 K using Langevin dynamics for temperature control; Md2 created a 1000 ns simulation of the whole unrestrained system, with SHAKE to perform hydrogen bond length constraints and no force evaluation for bonds with hydrogen, at constant pressure with isotropic pressure scaling and the with an average pressure of 1 atm, at a temperature of 310 K and Langevin dynamics for temperature control with a random seed. A leapfrog integrator was used, and a collision frequency of 1 ps^−1^. A total 500,000,000 MD steps were performed per simulation, with a time step of two femtoseconds. The total potential(EPTOT) and dihedral DIHED energy values obtained from the cMD simulation were used to calculate thresholds and to set up accelerated MD (aMD) runs [3]. Each aMD created a 1000 ns simulation of the system with a total of 500,000,000 MD steps with a time step of two femtoseconds, SHAKE for hydrogen bonds, and no force evaluation for bonds with hydrogen, a nonbonded cutoff of 8 Å, constant pressure and isotropic pressure scaling, simulated at 310 K using Langevin dynamics as a thermostat, with a leapfrog integrator, and a collision frequency set at 1 ps^−1^.

Simulations including the TIP4-D water model [13] were set up using the same protocol as described above for the different force field simulations. For the implicit solvent simulations, the starting structure was prepared with tLEaP to use the modified ff14SBonlysc as a force field without water box parameters. Several generalized Born (GB) implementations were tested, but only the GB8 solvation model was found to be potentially useful. After an initial minimization of 5000 maximum cycles and 2500 ncycles of steepest descent, the simulations were executed for a total of 1000 ns per run at a temperature of 310 K, using Langevin dynamics as a thermostat and a time step of two femtoseconds. The starting structure for the Histatin 5 simulations was created using PyMOL v1.8.6.0 [31] in an unstructured format to avoid conformational bias. The starting structure for c-MYC^1-88^ was created using the QUARK ab initio protein structure prediction software [32,33].

### 2.2. Trajectory Analysis

Trajectory clustering analysis was carried out with CPPTRAJ (AmberTools16 [34]), employing the k-means algorithm, a total of four clusters and a random initial set of point distribution. The Scatter Biosis software [35] was used to calculate the theoretical SAXS intensities of the simulation representative structures, for comparison with the experimental data. The experimental SAXS data were analyzed using GNOM and PRIMUS, part of a suite of applications developed for small angle scattering data analysis (ATSAS data analysis software) [36,37,38]. The theoretical Cα proton chemical shifts were calculated for each trajectory, sampled every 10 ns, using SPARTA+ [39]. For the MD data, secondary structure propensity calculations were carried out with VMD-Timeline [40]. The VMD calculation provides the total random coil, turn, α-helix and β-sheet values of each residue sampled during the MD trajectory. Of these, the values for the α-helical and β-sheet contents per residue were extracted and calculated as a percentage of the total secondary structure propensity for the trajectory. The α-helix similarity (*Sα*) assessment was conducted using the open-source PLUMED library version 2 [41], implementing Pietrucci and Laio’s (2009) protocol [42]. Principal component analysis (PCA) calculations were carried out with Bio3D [43]. Time-lagged independent component analysis (TICA) and associated plots were produced using the PyEMMA 2.5.7 package [44]. TICA was performed using the backbone torsion angles, which refers to all the backbone phi/psi angles, for featurization at a lag of 20 nanoseconds projected over two independent components. The radii of gyration of 45 structures per TICA state were considered to calculate the average. The trajectory analysis and plots were created with custom Python scripts. The elbow cluster validation method was implemented using the Yellowbrick package. The k-means clustering applied the k-means algorithm that is part of the sklearn.cluster package. K-means clustering was parameterized using the k-means++ initialization method, with a maximum iteration of 100,000 cycles and 100 cycles of k-means run with different centroid seeds. The peak minimum and maximum analysis of the radius of gyration over time plot was done using the argrelextrema package imported from the scipy.signal module. The local peaks were found using a window of 500 to avoid the noise of neighboring peaks. The monitoring of contact activity in the MD simulation trajectories was achieved using the Python module tagging.py from the Timescapes 1.5 suite of programs [45,46]. This application maps functionally important residues based on pairwise residue interaction during the trajectory. The cutoff contact distance was set at 6.0 angstroms. The turning.py module from Timescapes 1.5 was used to map residues based on their correlations of backbone pivot angles, which display hinge bending. The pivot angle coefficient calculations are based on the “pseudodihedral” angles created by four consecutive α-carbons [45].

### 2.3. Experimental Data

The Histatin 5 SAXS data were generated by Cragnell et al. (2016) [47], and the Histatin 5 NMR data were derived from Raj, Marcus and Sukumaran (1998) [48].

The c-MYC^1-88^ NMR-derived secondary structure propensities were obtained from Andresen et al. (2012) [49].

### 2.4. Markov Chain Monte Carlo

Markov chain Monte Carlo (MCMC) simulations were run with the PHAISTOS program package for protein structure inference [28]. Two sets of MCMC independent simulations were set up with 25 threads each. Each thread simulated a total of 2000 structures. Therefore, a total of 100,000 structures were obtained for analysis. To avoid any structural bias, the simulations were parameterized using the amino acid sequence as the sole input. The backbone and sidechains were sampled using both pivot-uniform and sidechain-uniform moves. These moves produce a random, uniformly distributed rotation of the dihedral (φ,ψ angles) and sidechain torsion angles (χ angles) in single residues. The energy terms integrated the Profasi force field, parameterized to simulate interactions in the presence of a solvent. MCMC produces a collection of samples from a dense stationary (*π*) target distribution. The Markov chain builds an equation in which new states are accepted or rejected based on the following probability:

(1) $$\pi \left(x\right)P\left(x\;\to \;x^{\prime }\right)=\pi \left(x^{\prime }\right)P\left(x’\;\to \;x\right)$$

The probability of inhabiting state *x* multiplied by the probability of going from state *x* to state *x*’ is reversibly equal to the probability of moving from state *x*’ to state *x*. The next phase is to define two transition probability steps—the proposal probability and the acceptance–rejection probability:

(2) $$P\left(x\;\to \;x^{\prime }\right)=Pp\left(x\;\to \;x^{\prime }\right)Pa\left(x\;\to \;x^{\prime }\right)$$

The proposal probability $Pp\left(x\;\to \;x^{\prime }\right)$ corresponds to the calculated probability of proposing a given state and the acceptance probability $Pa\left(x\;\to \;x^{\prime }\right)$ corresponds to the calculated probability of accepting the new state or rejecting it.

The Metropolis–Hastings algorithm was used as the acceptance criterion for the simulation method:

(3) $$P\left(x\;\to \;x^{\prime }\right)=min\left(1,\frac{\pi \left(x^{\prime }\right)Pp\left(x^{\prime }\to \;x\right)}{\pi \left(x\right)Pp\left(x\to \;x^{\prime }\right)}\right)$$

Assuming unbiased transitions, the algorithm fully accepts the new state if its probability is increased according to the target distribution ((*x*’) > π (*x*)). If the probability is lower, the acceptance will depend on how unfavorable the new conformation is. This ensures harmonious sampling and a congruent probability distribution, in which the structures are sampled according to their conformation favorability.

## 3. Results

MD simulation and convergence analyses were applied to the experimental data available for two human IDP protein models, Histatin 5 and the N-terminal domain of the human oncoprotein c-MYC (c-MYC^1-88^). Both proteins are known to be extensively intrinsically disordered, and the availability of published experimental data makes them excellent model systems for judging the quality of MD simulations based on various force field variants and water models.

### 3.1. Histatin 5

Histatin 5 is a human salivary protein, 24 amino acids in length, that is known for its antimicrobial and antifungal role. It has a completely unstructured conformation, which was experimentally characterized by small-angle X-ray scattering (SAXS) [47]. To compare the Histatin 5 SAXS data to the results obtained from the various MD simulations, a noise reduction method was first performed to address the complexity of the simulation trajectory using k-means clustering to identify the average structures for the most abundant states sampled during the simulation. Table 1 lists the radii of gyration (*R_g_*), a measure of protein compactness, for each representative structure calculated for the two most abundant k-means clusters for each force field variant tested.

**Figure 1:**
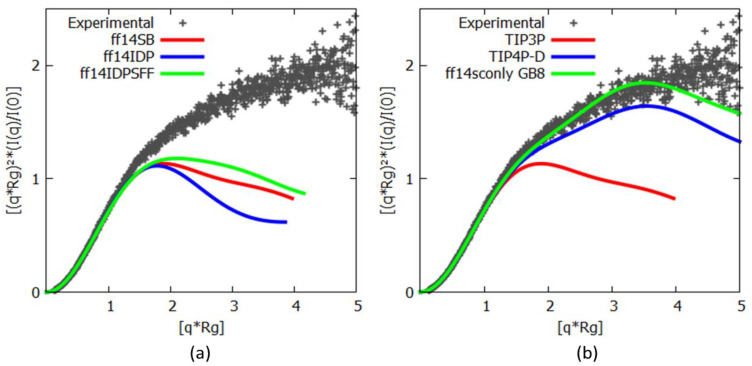
Kratky plots comparing the experimental small-angle X-ray scattering (SAXS) data (in gray) to representative structures obtained from each of the (**a**) simulated force field conditions: ff14SB and the modified ff14IDPs and ff14IDPSFF force fields and (**b**) solvation conditions: TIP3P, TIP4P-D and the implicit solvent GB8.

A comparison of the experimentally determined *R_g_* with the values derived from the simulations shows that the values do not agree. A similarly poor correlation is also evident from Kratky plots comparing the experimental data with curves calculated from representative structures modeled in the MD simulations (Figure 1).

The ff14SB force field—or either of the variants (ff14IDPs, ff14IDPSFF)—thus produced overly folded protein ensembles that are not consistent with the experimental data. Although ff14IDPSFF performs very slightly better than ff14SB in terms of reduced protein compaction, ff14IDPs performed significantly worse. With these force field variants failing to adequately describe Histatin 5, we next turn our attention towards different water models. Table 2 lists the *R_g_* values for each cluster centroid structure for the different MD trajectories as compared to the SAXS-derived *R_g_*.

These results reveal that the TIP4P-D and GB8 solvation modes create ensembles close to the experimental data. This is especially obvious when compared to the *R_g_* values obtained with TIP3P solvation. For simulations employing TIP4P-D, the two most abundant clusters consist of extended conformations, whereas the implicitly solvated GB8 simulations create conformations with a wider range of structure compactness, oscillating between averages of 10.68 Å and 14.14 Å (Table 2).

The conclusions from the comparison with the SAXS results are reiterated by NMR data based on a comparison between the calculated chemical shifts obtained from the MD trajectories and Histatin 5 NMR Cα proton chemical shifts. A comparison of the chemical shifts predicted from the various MD simulation trajectories makes it evident that the TIP3P water model is not a suitable choice for solvation, whilst both TIP4P-D and GB8 replicate the experimental findings accurately (Figure S1).

This confirms that water models play a substantially more influential role than any of the force field modifications tested earlier. Moreover, both TIP4P and GB8 implicit solvations appear to be capable of recreating the extended conformational nature of the Histatin 5 protein in conjunction with the standard force field AMBER ff14SB.

### 3.2. c-MYC^1-88^

While creating non-compacted ensemble structures for an IDP is a key requirement for any serious attempt to simulate such proteins computationally, it remains unclear from the Histatin 5 example shown above whether these conditions are capable of accurately recreating the transient secondary structure elements that are known to form spontaneously in larger IDPs. In order to test this, we turned to experimental data available for the N-terminal portion of the oncoprotein c-MYC.

**Figure 2:**
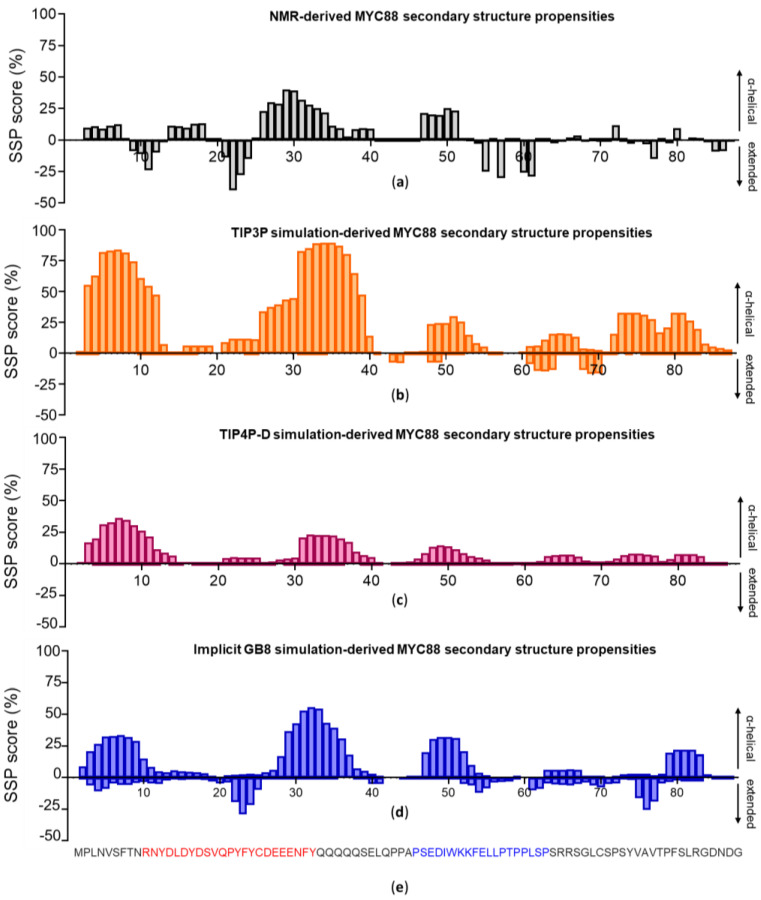
Comparison of (**a**) NMR-determined transient secondary structure propensities of c-MYC^1-88^ with (**b**) those obtained from the molecular dynamics simulations using the TIP3P solvation method, (**c**) those obtained from simulations using the TIP4P-D water model and (**d**) those obtained from the simulations using the implicit generalized Born (GB8) solvation method. The positive values on the *Y*-axis correspond to regions with a tendency to form α-helices, whilst the negative *Y*-axis values reflect regions with propensities towards extended structure formation. (**e**) Sequence of c-MYC^1-88^ with MYC-boxes MB-0 colored in red and MB-1 in blue.

The gene-specific transcription factor c-MYC regulates key aspects of cell proliferation and metabolic activity through controlling the expression of its target genes [50]. Apart from a structured “helix–loop–helix” DNA-binding domain located at the C-terminal end, the remainder of the protein (constituting ~70% of the overall primary amino acid sequence) is intrinsically disordered. Similar to the situation found in other intrinsically disordered proteins, a comparison of c-MYC orthologs among vertebrate species reveals only a moderate degree of sequence conservation, with the exception of five more highly conserved regions termed MYC boxes (MB-0 to MB-4). These MBs are thought to facilitate interactions between c-MYC and its numerous molecular partners, including transcriptional mediators, elongators and chromatin re-modeling complexes. Spanning the first 88 amino acids, c-MYC^1-88^ contains MYC boxes MB-0 and MB-1. Secondary structure propensities (SSPs) have been measured for this region by NMR [49] (Figure 2a).

Based on combined SSP and nuclear Overhauser effect (NOE) assessments, four main regions displaying transient ordered structure formation were identified: a β-turn at residues 21 to 24, helical regions comprising residues 26 to 34 and 47 to 54 and an extended region from residue 55 to 65. These assignments, specifying both the location of distinct secondary structure elements and the expected frequency of their appearance, provide us with a valuable guidepost to assess the accuracy of IDP simulation conditions.

In order to allow robust comparisons, the averaged secondary structure for each residue over the course of the entire simulation was calculated for each trajectory. We initiated our studies of c-MYC^1-88^ modeling with a combination of ff14SB with TIP3P, a standard set-up commonly used by many investigators to simulate folded proteins. As expected, this combination overestimates the frequency and boundaries of helical formations (Figure 2b). The experimental data indicate that there are no protein regions displaying more than 50% helical formation, highlighting the discrepancy between the simulation and the experimental results. The extended regions determined by NMR are also absent from the TIP3P simulation. The result essentially reiterates the conclusions previously drawn from the Histatin 5 simulations, namely that the TIP3P solvation method produces overly ordered and compact structures with an increased bias towards α-helical motifs.

We next employed the combination of ff14SB with TIP4P-D, which already looked promising earlier with Histatin 5. The TIP4P-D simulations bring the helical formation values more in line with the experimentally determined values. However, TIP4P-D samples the extended motifs insufficiently (Figure 2c). In contrast, the implicitly solvated simulations replicates the extended formations between residue 21 and 25 congruently, as well as the α-helical regions formed by residues 26 to 34 and 47 to 54 (Figure 2d). Although the implicitly solvated simulation creates overly ordered loci at the two terminal regions, it otherwise displays a noteworthy degree of consistency with the experimentally determined secondary structure propensities. It should also be considered that the c-MYC^1-88^ used in the NMR study still contained an N-terminal oligo-histidine tag, which may have had a (minor) influence on the dynamics of the immediately adjacent N-terminal helices [49]. The overall conclusion is affirmed by the total values for helical and extended β-sheet contents, which demonstrates that both explicit water models replicate the helical content well, but only GB8 recreates the extended portions predicted by experiment (Figure S2).

### 3.3. Assessing Convergence

**Figure 3:**
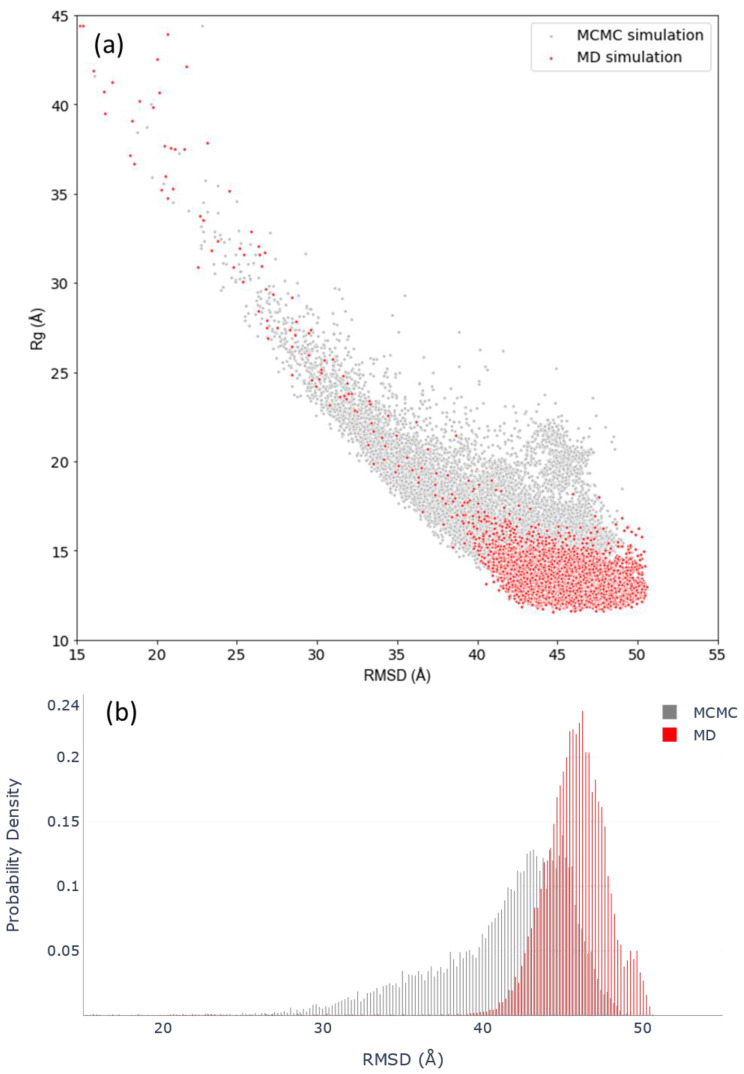
(**a**) Conformational landscape of c-MYC^1-88^ determined by Markov chain Monte Carlo (MCMC) versus MD GB8 simulations. (**b**) Frequency distribution of the root mean square deviation (RMSD) values for MCMC and MD GB8 simulations.

From this point onwards, the analyses will focus on results obtained with the GB8 implicit solvation simulations. To compare the conformational landscapes derived from MCMC and MD simulations, both were defined in terms of the RMSD and *R_g_* of the structural ensembles generated. Although a correlation between experimental and modeled structures in terms of their secondary structure elements was already shown above, we wanted to develop additional metrics for assessing the compactness, flexibility and conformational divergence of the c-MYC^1-88^ data. The RMSD was calculated against a completely extended structure as a reference. Therefore, the highest RMSD and low *R_g_* values correspond to structures in folded states and, conversely, the lower RMSD and high *R_g_* values correspond to the most extended structures. The MCMC landscape and RMSD frequency distribution plot indicate that the most probable states, and most well-sampled conformations, occupy the highest *RMSD*. This correlates with the lowest *R_g_* values, between 13 and 18 Å, hinting at c-MYC^1-88^ preferentially inhabiting a series of relatively compact states, whereas the MCMC probability landscape also identifies a wealth of extended c-MYC^1-88^ states (Figure 3).

**Figure 4:**
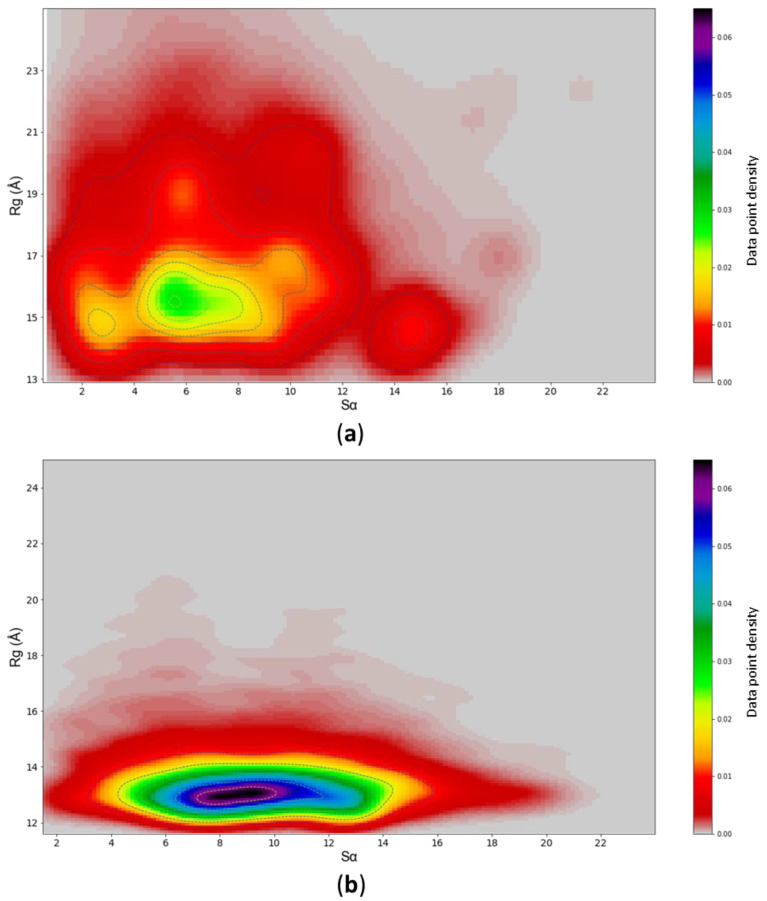
(**a**) MCMC conformational landscape of c-MYC^1-88^ defined in terms of its α-helix similarity (Sα) and *R_g_*. (**b**) The same for MD GB8 simulations.

The rationale for creating such a landscape is not to directly assess the protein dynamics of c-MYC^1-88^ from it (MCMC simulations do not reflect a temporal progression of a system, but rather give an overview of the range of possible conformational spaces available to the protein (Figures S3 and S4). A comparison of the MCMC and MD conformational landscapes results calculated in the same manner shows that the two methods create structures that inhabit an extensively overlapping landscape, especially when referring to the most compact conformations. Furthermore, using *Sα*—a metric of α-helical content similarity—as a conformational descriptor against the *R_g_*, allows for investigation into the helical sampling of the MD landscape; the MD simulation explores a wide range of helical content, similar to the range explored by the MCMC landscape (Figure 4).

**Figure 5:**
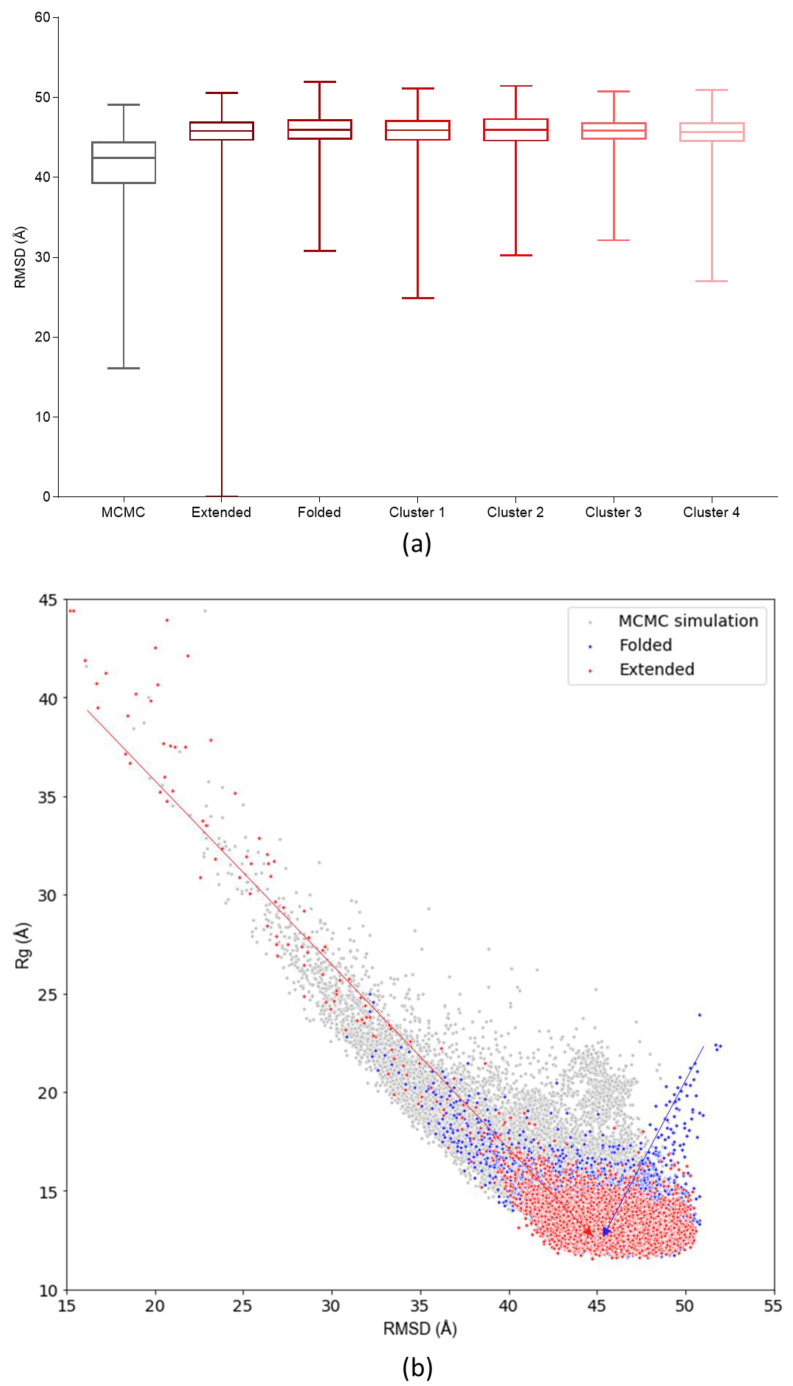
(**a**) Boxplots comparing the MCMC RMSD values of the MD simulations parameterized with different starting structures: “Extended” corresponds to fully unstructured initial coordinates; “Folded” to a structure created with an ab initio software; the “Cluster” structures correspond to the different k-means centroids. (**b**) MCMC conformational landscape compared to MD of c-MYC^1-88^ simulations starting from fully extended initial coordinates (in red), or from a folded conformation (in blue). The arrows highlight the shortest path from the starting point to the equilibrated conformational pool.

From these data, it is evident that the MD simulations do not become trapped in overly helical states, but sample within a wide basin of *Sα* values. Nevertheless, the MD simulations preferentially explore the lowest *R_g_* states, which could be due to a variety of reasons. It is possible that the most extended states, as predicted by the MCMC simulation, are not very favorable energetically. Alternatively, the MD simulations may require more extensive sampling on a longer time scale. To assess the validity of these two hypotheses, MD simulations were repeated, starting with the coordinates from an extended, compacted structure or MCMC k-means clustering centroids as starting points (Figure S4). The results demonstrate that the choice of initial structures has no significant impact on the MD simulation conformational sampling range; all simulations converge on the same common space (Figure 5).

Given that our finally selected simulation parameterization (ff14SB/GB8) is in agreement with experimentally determined secondary structure propensity—and the starting coordinates do not bias the simulations towards more compact structures—any differences between the MCMC and MD simulations are therefore more likely to be caused by the energy functions used to calculate the structural properties, rather than any bias introduced by the starting point of the simulation.

### 3.4. c-MYC^1-88^ Trajectory Analysis and Structural Insights

Plotting different trajectory analysis metrics of c-MYC^1-88^ suggests that the resulting landscapes do not contain any differentiated clusters (Figure S5). Such a lack of discernible clusters makes it difficult to deploy clustering algorithms to gain insight into the different molecular macrostates. K-means clustering and principal component analysis (PCA) reveal little or no distinct divisions between the different structural states (Figures S6 and S7; Table S1). Even the description of conformational space based on *Sα* shows that the helicity of the protein is noisy and rapidly changing (Figure S8).

**Figure 6:**
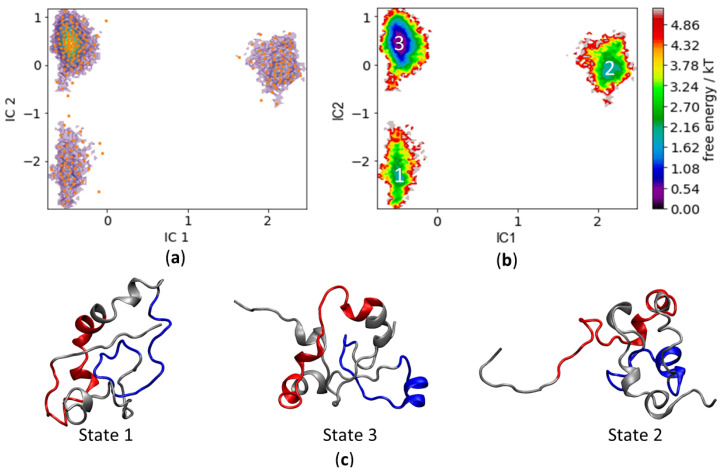
(**a**) Free energy plot showing the conformational basins with the k-means overlapped cluster centers (orange). (**b**) Free energy surface plot of the energy basins identifying the protein metastable states. (**c**) The representative structures for each basin, highlighting the location of MB-0 (red) and MB-1 (blue).

Time-lagged independent component analysis (TICA) is a linear transformation method oriented towards finding coordinates of maximal correlation given a time lag. Similar to PCA, the slowest motions, rather than maximal amplitude motions, are tracked [44]. The main advantage of TICA over PCA is its lower dependence on the distance metrics since TICA is not concerned with the variance of atomic displacement, but rather with the speed of temporal change embedded into the process of structural change. However, the featurization of data remains important and must be considered to minimize statistical error [51]. The data were projected over two dimensions (the first two independent components [ICs]) with a lag of 20 nanoseconds. The two ICs correspond to the two slowest transitions in the data set. Plotting the two ICs as a free energy plot shows that TICA creates a landscape with well-defined and separated minima basins that are less prone to clustering errors (Figure 6).

The three conformational basins identified by TICA correspond to three metastable states. K-means clustering can now be used to discretize the IC landscape to allocate trajectory structures to their respective k-means clusters, which allows the clustered microstates to be assigned to the three metastable macrostates. The Perron-cluster cluster analysis (PCCA++) method was used to extract the representative structures for each macrostate.

The main long-lived basin, corresponding to the most abundantly visited pool of conformations, is State 3 (Figure 6b,c). This is a key finding, as this pool of conformations affords a window of opportunity for drug discovery—it is a frequently visited protein state and at the heart of the two slowest transition processes. Exploring along the IC1, it can be easily determined that the slowest process corresponds to the transition between State 3 and State 2. It is interesting to note that the structural dynamics from State 3 to State 2 entail the extension of the extreme N-terminus, which includes MB-0, from the main compacted body of c-MYC^1-88^. The second slowest process along the IC2 axis identifies the transition between State 3 and State 1, which moves c-MYC^1-88^ to a more compact conformation. Overall, the TICA landscape identifies three c-MYC^1-88^ metastable states: a very abundant pool of conformations (State 3), averaging 12.96 Å in *R_g_*, followed by the slowest transition to State 2, which mainly consists of the N-terminal extension—with an average *R_g_* of 13.3 Å. The transition from State 3 to State 1 is the second slowest process, in which the molecule acquires a slightly more compact structure, with an average *R_g_* of 12.7 Å.

**Figure 7:**
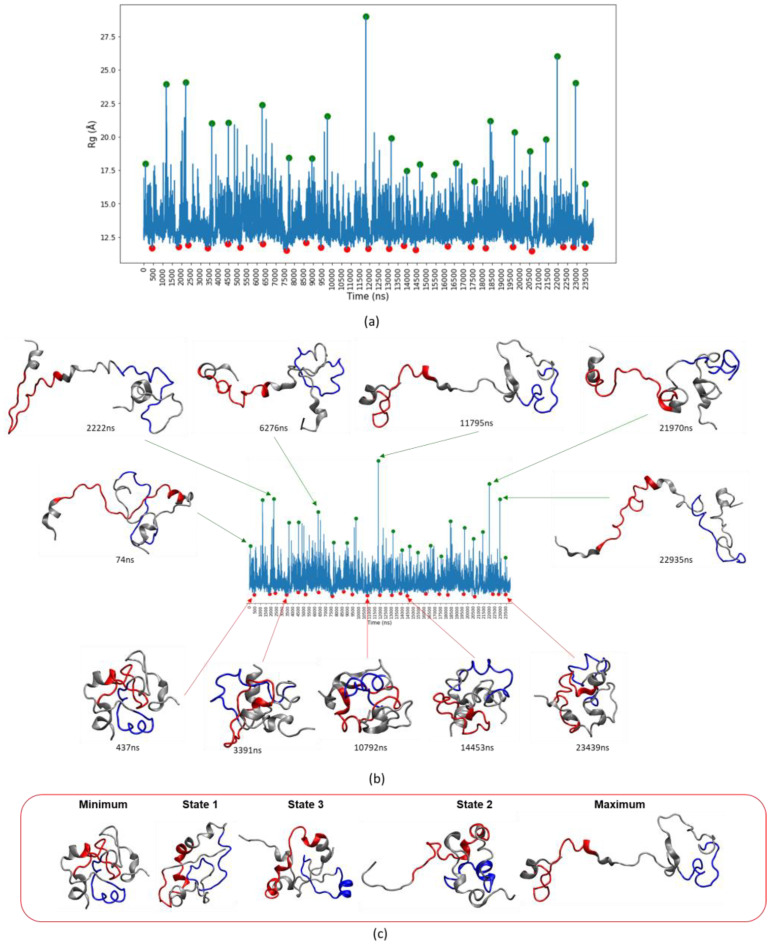
(**a**) The *R_g_* values over the course of the trajectory with the identified minima and maxima (identified by red and green dots, respectively). (**b**) Examples of some of the minima and maxima conformations over time. MB-0 is highlighted in red and MB-1 in blue. (**c**) Representative structures of c-MYC^1-88^. The “minimum” and “maximum” structures were derived from the *R_g_* peak detection and were combined with the three states that correspond to the three time-structure independent components analysis (TICA) metastable states.

For IDPs such as c-MYC^1-88^, it is still crucial to assess the highest amplitude motions because they usually correspond to rarer but still important protein conformations. As previously established, PCA is not a useful conformational space descriptor method, and a new strategy must be implemented. The strategy presented here goes back to basic geometric measures, in this case *R_g_*, to track minima and maxima over time (Figure 7a).

The maxima correspond to the most extended and the minima to the most compact structures (Figure 7b). The results emphasize that all of the most extended structures of c-MYC^1-88^ involve the extension of its N-terminus. A sequence of structural snapshots summarizes the range of protein conformations available to c-MYC^1-88^ (Figure 7c). The three TICA states correspond to a well-sampled pool of conformations, with especially State 3 being most abundant. State 3 represents an intermediate conformation that can become more compact—and therefore less likely to interact with molecular partners—or project the N-terminus containing MB-0 outwards in an extended conformation (the “fly-casting” extension), which may help to promote binding with molecular partners.

**Figure 8:**
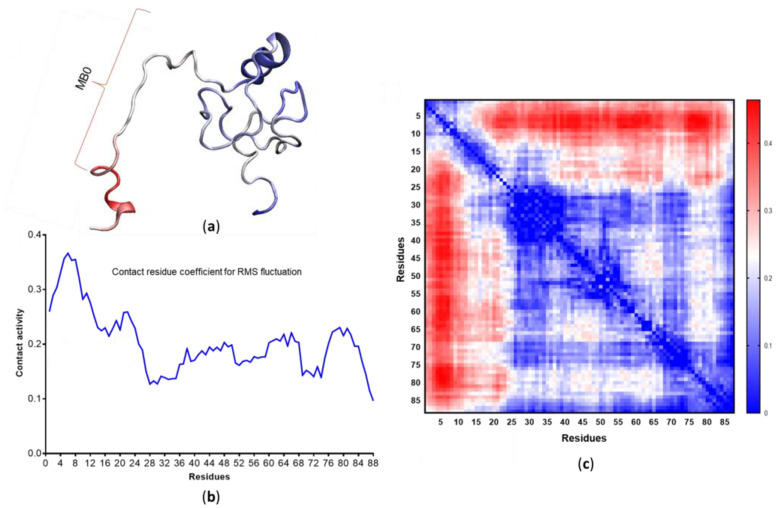
(**a**) Contact activity data superimposed on a representative structure. (**b**) Linear graph containing the contact residue coefficient for root mean square fluctuation per residue. (**c**) Heat map identifying active versus inactive protein regions according to their contact activity coefficient—blue identifies areas of stability and red identifies areas of increased activity.

The structural dynamics of MB-0 are especially intriguing because MB-0 corresponds to a separate and independent transactivation domain [52]. The results reported here are further supported by contact data identifying residues 1 to 24 as displaying the highest contact formation and breaking activity (Figure 8).

**Figure 9:**
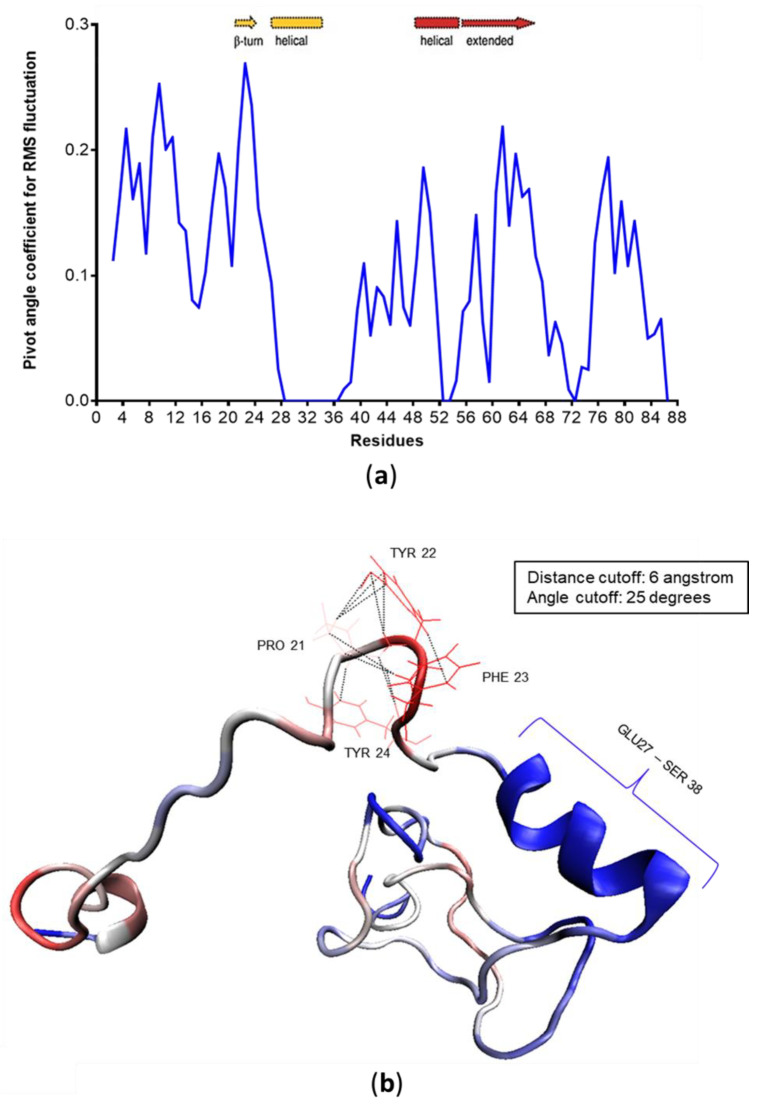
(**a**) Linear data for the pivot angle coefficient (residues involved in the formation of pivot angles). The same data are mapped onto a representative structure (**b**). The most prominent pivot angle is the β-turn formed by amino acids 20 to 24, which corresponds to the extended region identified by NMR [49].

In contrast, the remainder of the protein is structurally comparatively stable. Additionally, calculations of the pivot angle formation coefficients identify residues 20 to 24 as the main hinge of c-MYC^1-88^ (Figure 9). The hinge is formed by the same residues predicted by NMR data (Figure 2a) to form a β-turn. Other notable pivot angles that contribute to the N-terminal flexible nature include residues 8 to 12, which allow the extreme N-terminus to extend further away from the rest of the protein. A stable helix spanning residues 27 to 38, consistent with NMR observations, creates a divider that separates MB-0 and MB-1 from each other, and allows them to act autonomously in terms of their structural dynamics. The ordered region, acting as a divider, allows MB-0 to perform its alternating extension and compaction motions, fly-catching its molecular partners, without disrupting the local conformation of MB-1. As seen from the network of contacts (Figure 8; Figure S9), MB-1 and the phosphodegron residues are crucial hubs in the system, making it essential for that protein region to remain more structurally stable than MB-0.

## 4. Discussion

IDPs are challenging to characterize on a structural and a functional level. Many experimental methods (especially structural techniques), that have been used successfully for decades to study globular proteins, fall short when it comes to applying them to IDPs. The problems with acquiring structural information about IDPs significantly impede other types of functional investigations (such as site-directed mutagenesis approaches) because biochemists lack models suitable for formulating testable hypotheses.

Especially during the last decade, MD simulations have established themselves firmly as an essential method to gain a deeper understanding of structural—and especially dynamical—aspects of macromolecular systems. Standard MD parameterization methods (force fields and water models) are predominantly based on the structures of folded protein available in databases and therefore are of limited value for simulating IDPs. Such biases include increased helical content and tendencies to fold proteins into structures that are too compact. Due to this, novel MD methods have been developed to improve the accuracy of simulations of IDPs. Here, different approaches were tested and benchmarked against SAXS- and NMR-derived data. These approaches included force field re-parameterizations and the implementation of novel solvation methods. Using Histatin 5 and c-MYC^1-88^ as IDP model systems revealed that the modified force fields tested did not perform as accurately as expected when compared to the available experimental data. None of the force field variants specifically developed for IDP simulation managed to generate the random coil extended Histatin 5 conformers detected by the SAXS data. In contrast, modified water models showed more promise. The explicit solvation TIP4P-D and the implicit GB8 outperformed TIP3P. With TIP3P solvation, certain regions of c-MYC^1-88^ displayed nearly 100% helical content—a completely stable secondary structure element—which deviates from the NMR estimations [49]. Despite some discrepancies between c-MYC^1-88^ N-terminal NMR SSP and the simulated equivalents, the modified solvation methods, TIP4P-D and GB8, brought the helical motifs into a much more realistic agreement with the NMR SSP data in terms of propensity and location along the primary sequence. Although the TIP4P-D model was excellent in terms of reproducing the transient α-helical structures, it failed to reproduce the β-sheet extended regions that were also detected experimentally [49]. Of all the tested methods, GB8 implicit solvation turned out to be the most balanced method in terms of reproducing the α-helical and β-sheet propensities at the anticipated loci (Figure 2, Figure S2).

Another contentious topic, especially when it comes to simulations of IDPs, is the convergence criteria of simulation data. An objective demonstration—showing that an MD simulation samples a varied conformational space adequately without getting trapped in local minima—is as crucial as it is challenging. Here, we present a new approach that compares the conformational ensemble produced by MDs to a structural landscape (defined by RMSD and *R_g_*, or *Sα* and *R_g_*) derived from an MCMC probability distribution. Our results show that, irrespective of the starting structure (compact/extended), the MCMC and MD simulations converge on an overlapping conformational space. When comparing the MCMC data to the MD results, it becomes apparent that both preferentially sample the compact states. However, MCMC predicts the existence of highly extended states that are not sampled expediently by MD. This suggests that the most extended conformations, although possible from a probabilistic point of view, represent rare and energetically unfavorable states. Thus, MD simulations systematically sample the collections of structures from the predicted landscape that are structurally and dynamically most relevant.

Histatin 5 is an interesting model system in its own right, but is especially suited for assessing the performance of force field variants and modified solvation systems in simulating a highly unfolded structure lacking discernible secondary structure elements. The major goal of the work described here, however, was to gain new insights into the structural properties of c-MYC^1-88^. The fact that the combination of ff14SB with the GB8 implicit solvation method reproduces the secondary structure elements identified by NMR studies most faithfully suggests that at least certain structural aspects of this IDP are also reflected in the MD trajectories. Despite the overall high mobility of c-MYC^1-88^, a detailed analysis suggests the presence of three distinct clusters of states. We identified a compact state engaged in a variety of internal interactions, which appears less likely to establish any intermolecular interactions with other proteins. We also detected a state where the N-terminus (encompassing residues 1 to 24), including MB-0, extended away from the remainder of the protein, which is compatible with the concept that MB-0 may be involved in a “fly-catching” motion of molecular partners. This ability to extend is facilitated by hinges spanning residues 20 to 24 (also corresponding to a β-turn detected by NMR). The MD results also show that a region identified as having significant α-helical propensities (residues 27 to 38) may act as a stabilizing anchor, allowing MB-0 to extend without affecting local structures formed around MB-1. Our observations thus support the role of MB-0 acting as a structurally distinct and independent transcriptional transactivation domain (TAD). In contrast to the identified MB-0 motion, the simulations predict that MB-1 remains more stable throughout the simulation. This finding, taken together with the elucidation of the protein’s most abundant and long-lived TICA metastable state, afford a window of opportunity for drug discovery and for reaching a better understanding of the important phosphodegron region contained within MB-1. The three representative structures can be used for further enquiry into ligand docking and in silico compound screening.

## 5. Conclusions

For a protein such as c-MYC^1-88^, where the location, dynamics and structural aspects of the functional domain(s) are still poorly characterized, such insights provide a basis for more detailed site-directed mutagenesis approaches to establish structure/function links and to assess the “druggability” of these key functional domains. Furthermore, the methods presented here provide a clear strategy for the simulation optimization, convergence assessment and trajectory analysis that are equally applicable to simulating other IDP systems that are currently often left unstudied due to a lack of suitable analytical methods.

## Figures and Tables

**Table 1:** Comparison of the radius of gyration (*R_g_*)values for each representative structure (cluster and simulated force field condition) with the experimentally determined *R_g_* of Histatin 5.

Force Fields	Cluster 1	Cluster 2	Experimental
ff14SB	9.15 Å	7.71 Å	
ff14IDPs	7.38 Å	8.15 Å	13.8 Å
ff14IDPSFF	7.48 Å	9.87 Å	

**Table 2:** Comparison of the *R_g_* values for each representative structure (cluster and simulated force field condition) with the experimentally determined *R_g_* of Histatin 5.

Water Model	Cluster 1	Cluster 2	Experimental
TIP3P	9.15 Å	7.71 Å	
TIP4P-D	13.47 Å	12.12 Å	13.8 Å
Implicit GB8	10.68 Å	14.14 Å	

## Supplementary Materials

The following are available online at https://www.mdpi.com/2075-1729/10/7/109/s1, Figure S1: Comparison of NMR-determined HA chemical shifts to calculated chemical shifts from the simulation trajectories; Figure S2: Comparison of the helical and extended SSPs between the NMR-determined values and the values predicted from the explicit TIP4P-D and the implicit GB8 solvation models; Figure S3: Comparison of (a) NMR-determined transient secondary structure propensities of c-MYC^1-88^ with (b) those obtained from the MCMC simulation; Figure S4: MCMC landscape of c-MYC^1-88^ colored by K-means cluster; Figure S5: Normalized MYC88 landscapes obtained by plotting RMSD values against different simple MD simulation metrics; Figure S6: c-MYC^1-88^ RMSD/Rg plot depicting the clusters and centroids; Figure S7: PCA plot depicted with a kernel density estimation heatmap for detection of areas with high density of data points; Figure S8: *Sα* evolution over time; Figure S9: Contact activity heatmaps; Table S1: Eigenvalues, explained variance and cumulative explained variance for the first 6 principal components.
