# Supplementary material for: Optimization of Molecular Dynamics Simulations of c-MYC1-88—An Intrinsically Disordered System
Source: Life (Basel). 2020 Jul 10;10(7):109. doi: 10.3390/life10070109 (PMC7400636; doi:10.3390/life10070109)
Supplement: Supplementary file 1 [file life-10-00109-s001.pdf]

life

Supplementary Materials:

Histatin 5 NMR HA chemical shifts compared to the calculated chemical shifts for the different trajectories (Figure S1). Figure S1 shows that the TIP3P solvation model does not adequately replicate the experimental shifts, and this is further corroborated by the Mann-Whitney test p-values. The RMSE score calculated between the experimental and simulated data shows that the GB8 solvation solution is closest to the experimental chemical shifts – 0.12 ppm, while TIP4P-D and TIP3P achieved a RMSE of 0.14 and 0.21 ppm, respectively.

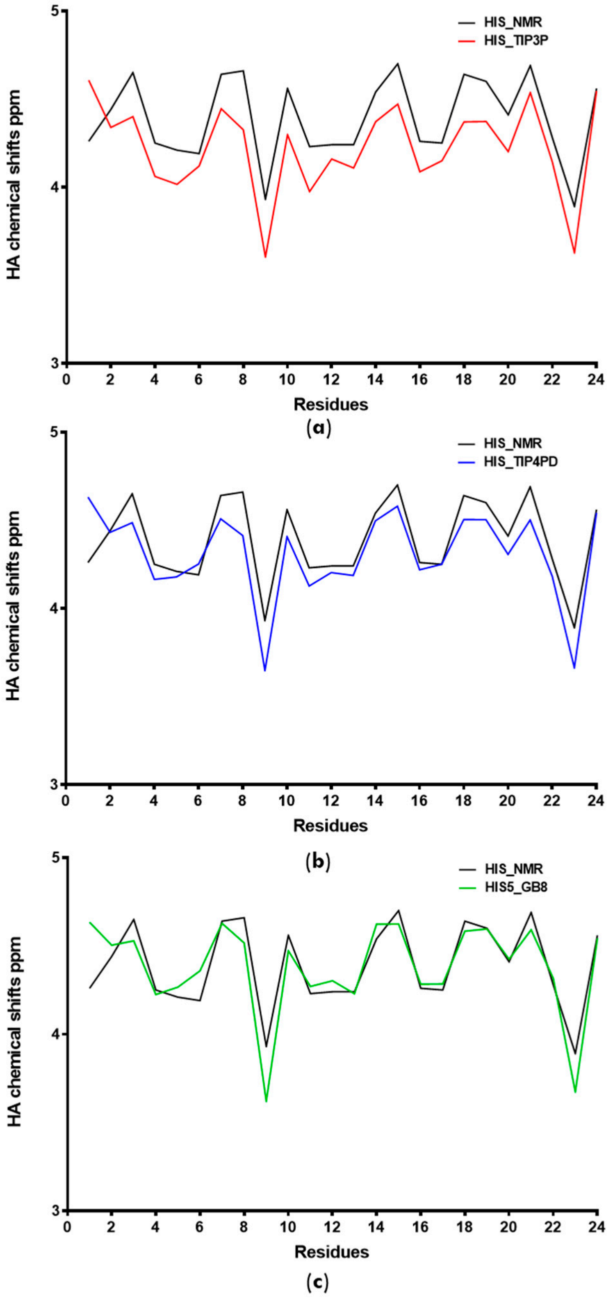

Figure S1. Comparison of NMR-determined HA chemical shifts to calculated chemical shifts for the simulation trajectories (**a**) using the TIP3P solvation method (p-value 0.0208), (**b**) using the TIP4P-D water model (p-value 0.1387) and (**c**) obtained from the simulations using the implicit GB8 solvation method (p-value 0.9874).

Figure S2 compares the total secondary structure content created by the trajectories with the ones obtained by NMR. Both GB8 and TIP4P-D accurately replicate the helical content of c-MYC[1-88], but TIP4P-D underestimates β-sheet propensities (p-value <0.0001).

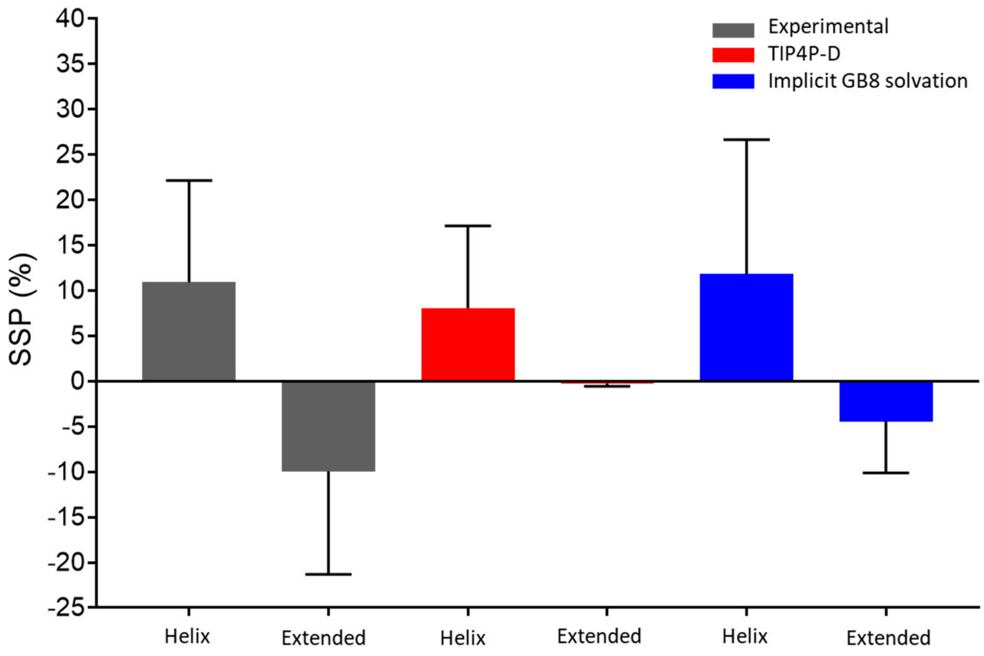

Figure S2. Comparison of the helical and extended SSPs between the NMR-determined SSP and the SSP values predicted from the explicit TIP4P-D and the implicit GB8 solvation models.

Figure S3 shows the comparison of the NMR-determined SSP to the MCMC-derived landscape showing that MCMC results are also mostly consistent with experimental data.

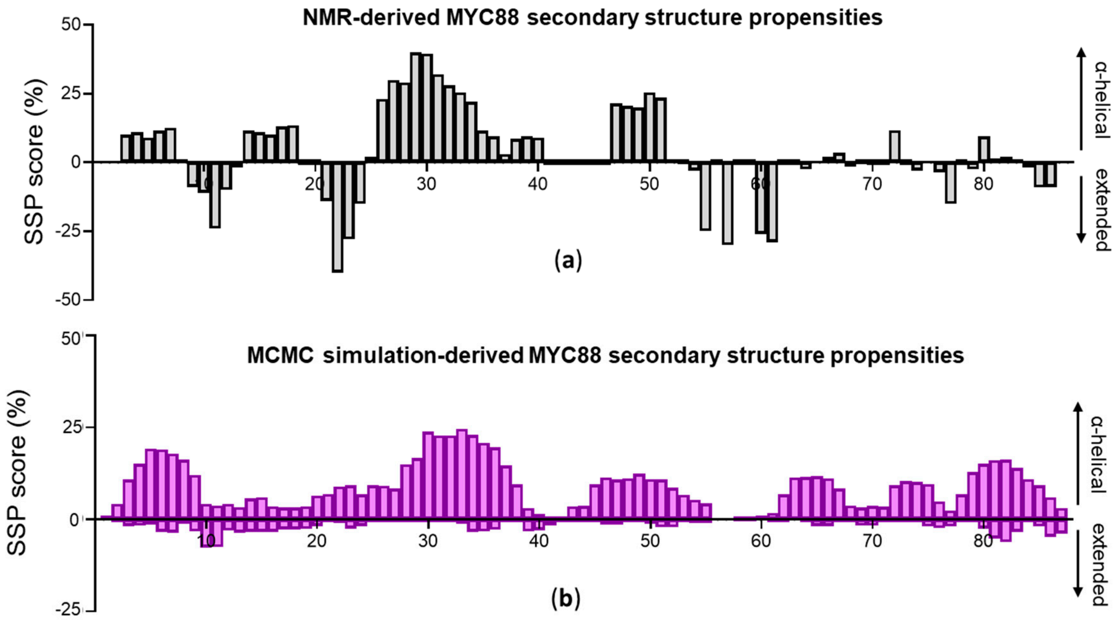

Figure S3. Comparison of (**a**) NMR-determined transient secondary structure propensities of c-MYC[1-88] with (**b**) those obtained from the MCMC simulation.

To assess the range of configurations being predicted by the MCMC distribution the K-means algorithm was used to reveal the average representative structures for a total of four conformational states (Figure S4).

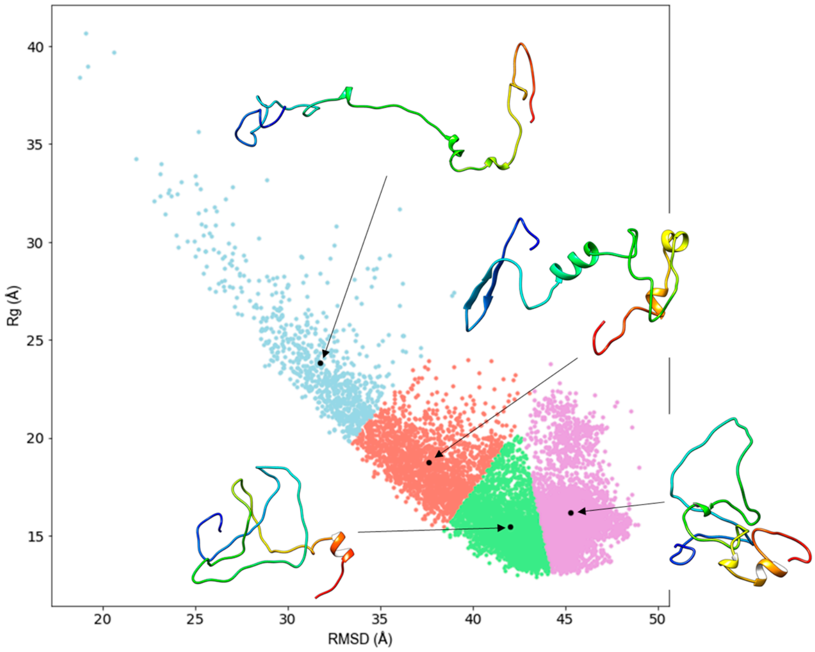

Figure S4. MCMC landscape of c-MYC$^{1\text{-}88}$ colored by K-means cluster. The representative average structures for each cluster correspond to the cluster centroids. The representative MCMC structures, defining MYC88's conformational range, were used as MD simulation starting coordinates to test for sampling optimization.

Figure S5 shows several descriptive landscapes obtained by plotting different trajectory analysis metrics against the RMSD, showing that the landscape does not contain any differentiated clusters.

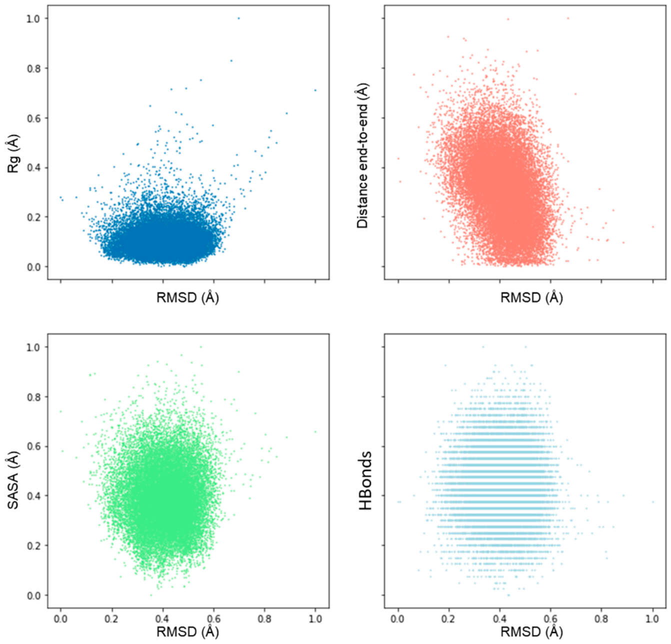

Figure S5. Normalized MYC88 landscapes obtained by plotting RMSD values against different simple MD simulation metrics: radius of gyration (Rg), the molecule's distance from N-terminal to the C-terminal (Distance end-to-end), solvent-accessible surface area (SASA) and the number of hydrogen bonds (Hbonds).

None of the landscapes in Figure S5 is 'clusterable', however clustering was still ~~be~~ attempted for the RMSD-R_g landscape to show how randomly the K-means clustering partitions the data, demonstrating that there is no real separation between the clusters (Figure S6).

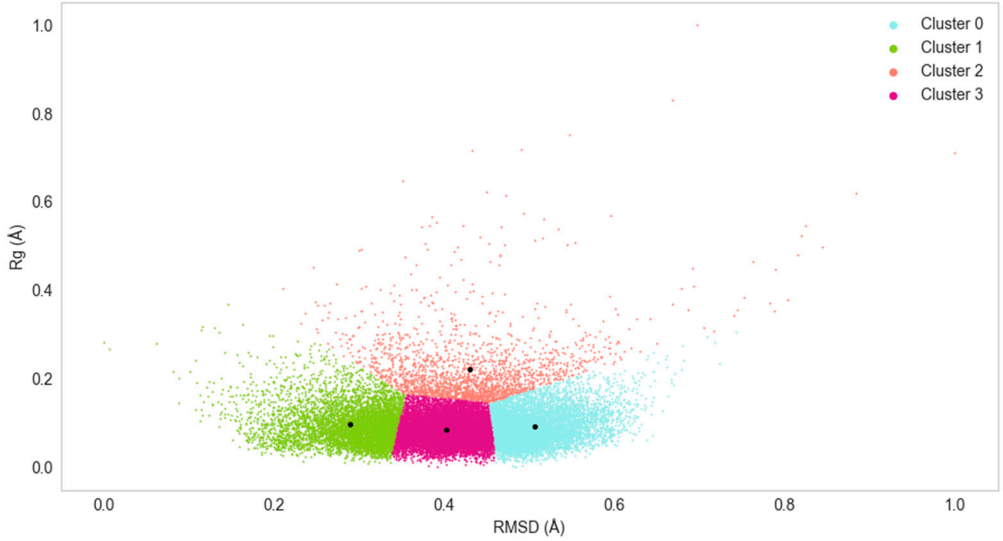

Figure S6. c-MYC$^{1-88}$ RMSD/Rg plot depicting the clusters and centroids calculated by the K-means algorithm for k=4.

PCA is another method of choice for the reduction the dimensional space. Figure S7 depicts the PCA landscape obtained from considering C-alpha atoms XYZ coordinates of c-MYC$^{1\text{-}88}$ over the course of the trajectory, projected over the first two principal components (PC) after applying kernel density estimation (KDE) to the data.

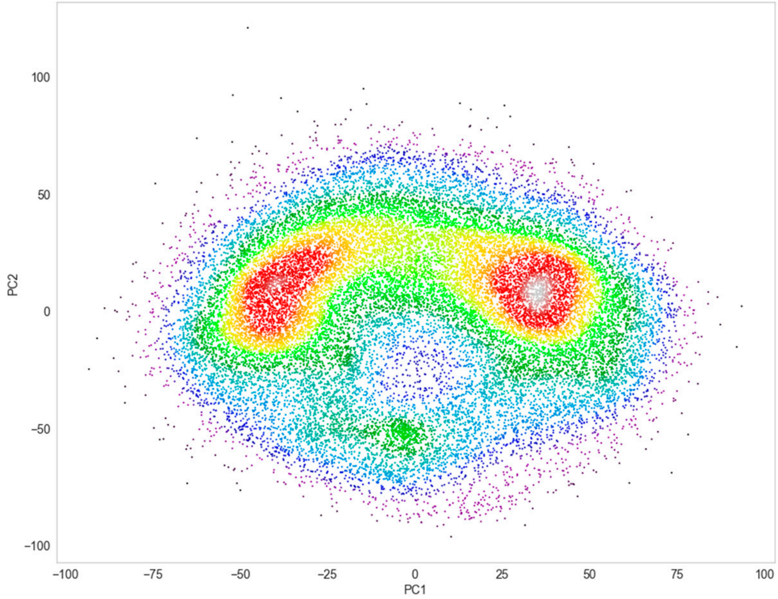

Figure S7. PCA plot depicted with a kernel density estimation heatmap for detection of areas with high density of data points.

With the KDE applied to the PCA landscape, the presence of two large clusters and a third smaller one at the bottom becomes evident. The first two PCs cumulatively explain only ~22.5% of the data variance. (Table S1).

Table S1. Eigenvalues, explained variance and cumulative explained variance for the first 6 principal components.

	Eigenvalue	Variance (%)	Cumulative (%)
PC 1	1273.756	12.328	12.328
PC 2	1042.172	10.087	22.415
PC 3	799.842	7.741	30.156
PC 4	719.519	6.964	37.120
PC 5	612.431	5.927	43.047
PC 6	513.945	4.974	48.022

Even the first 6 PCs cumulatively explain less than 50% of the data which is insufficient to derive any truly representative conclusions from the data.

Figure S8 presents the Sα evolution over time for the TIP4P-D and GB8 simulations. It shows how both simulations alternate very rapidly between configurations with varying helical content.

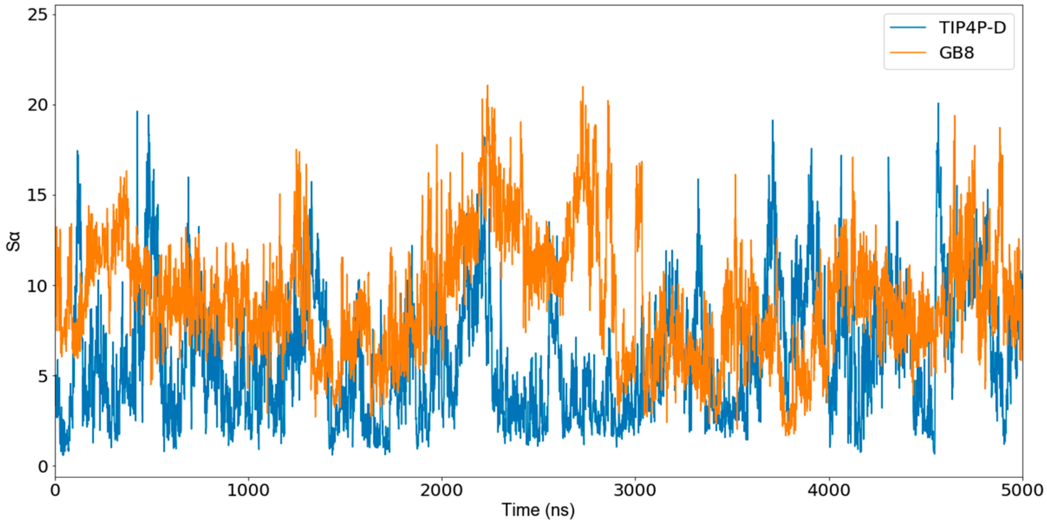

Figure S8. Sα evolution over the course of 5 independent and concatenated MD trajectories for TIP4P-D (in blue) and GB8 (in orange).

Figure S9 shows a side comparison of GB8 and TIP4P-D contact map. Interestingly, for the TIP4P-D simulations the C-terminus is more dynamic than predicted by the GB8 simulations.

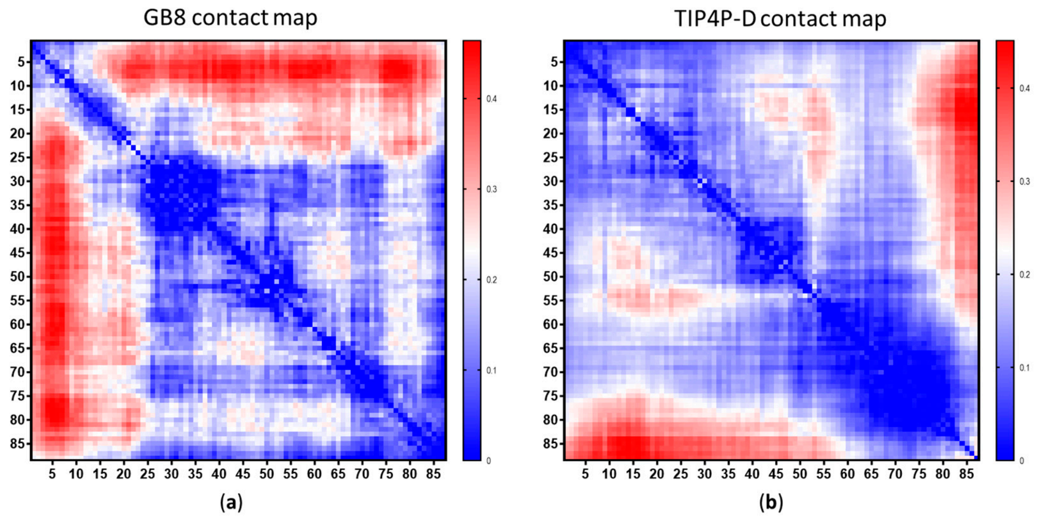

Figure S9. Contact activity heatmaps highlighting active and inactive regions of c-MYC$^{1\text{-}88}$ for (**a**) the GB8 simulations and (**b**) the TIP4P-D simulation.

© 2020 by the authors. Submitted for possible open access publication under the terms and conditions of the Creative Commons Attribution (CC BY) license (http://creativecommons.org/licenses/by/4.0/).
